# Material Performance Evaluation for Customized Orthoses: Compression, Flexural, and Tensile Tests Combined with Finite Element Analysis

**DOI:** 10.3390/polym16182553

**Published:** 2024-09-10

**Authors:** Daniela Trindade, Rachel Habiba, Cristiana Fernandes, André A. Costa, Rui Silva, Nuno Alves, Rui Martins, Cândida Malça, Ricardo Branco, Carla Moura

**Affiliations:** 1 Center for Rapid and Sustainable Product Development (CDRSP), Polytechnic of Leiria, 2430-028 Marinha Grande, Portugal; rachel.d.habiba@ipleiria.pt (R.H.); cristiana.h.fernandes@gmail.com (C.F.); rui.d.silva@ipleiria.pt (R.S.); nuno.alves@ipleiria.pt (N.A.); candida@isec.pt (C.M.); 2 Applied Research Institute, Polytechnic Institute of Coimbra, Rua da Misericórdia, Lagar dos Cortiços, S. Martinho do Bispo, 3045-093 Coimbra, Portugal; 3 Abel Salazar Biomedical Sciences Institute (ICBAS), University of Porto (UP), Rua de Jorge Viterbo Ferreira, No. 228, 4050-313 Porto, Portugal; 4 Department of Mechanical Engineering, University of Coimbra, Rua Luis Reis Santos, 3030-788 Coimbra, Portugal; 5 CIPER, Faculdade de Motricidade Humana, Universidade de Lisboa, 1495 Cruz Quebrada Dafundo, 1649-004 Lisbon, Portugal; 6 Associate Laboratory for Advanced Production and Intelligent Systems (ARISE), 4050-313 Porto, Portugal; 7 UNIDEMI, Department of Mechanical and Industrial Engineering, Nova School of Science and Technology, Universidade NOVA de Lisboa, Campus de Caparica, 2829-516 Caparica, Portugal; rfspm@fct.unl.pt; 8 Laboratório Associado de Sistemas Inteligentes (LASI), 4800-058 Guimarães, Portugal; 9 Coimbra Institute of Engineering (ISEC), Polytechnic Institute of Coimbra, Rua Pedro Nunes, Quinta da Nora, 3030-199 Coimbra, Portugal; 10 CEMMPRE-ARISE, Department of Mechanical Engineering, University of Coimbra, Rua Luis Reis Santos, 3030-788 Coimbra, Portugal; ricardo.branco@dem.uc.pt; 11 Research Centre for Natural Resources Environment and Society (CERNAS), Polytechnic Institute of Coimbra, Bencanta, 3045-601 Coimbra, Portugal

**Keywords:** customized orthoses, additive manufacturing, polymeric materials, ankle–foot orthosis, mechanical properties, static conditions

## Abstract

Orthoses are commonly used for treating injuries to improve the quality of life of patients, with customized orthoses offering significant benefits. Additive manufacturing, especially fused deposition modelling, enhances these benefits by providing faster, more precise, and more comfortable orthoses. The present study evaluates nine polymeric materials printed in horizontal and vertical directions by assessing their performance through compressive, flexural, and tensile tests. Among all materials, polycarbonate, polylactic acid, and ULTEM^TM^ 1010 showed the most promising results, not only because they had the highest mechanical values, but also due to their minimal or no difference in performance between printing directions, making them advantageous in orthoses fabrication. Based on this, a finite element model of an ankle–foot orthosis was developed to simulate the deformation, strain, and stress fields under static conditions. The findings aim to optimize material selection for orthotic fabrication, where ULTEM^TM^ 1010 is presented as the material with improved performance and durability.

## 1. Introduction

Orthopaedic devices are commonly used for treating injuries that can be caused by falls, age-related illnesses, or accidents. Orthoses are a type of assistive device that can be used in patients with physical impairments. The main function of these devices is to provide support and correct a certain segment of the body, confine joint movement, and minimize the risk of malformations by distributing the loading forces [1,2]. They can be categorized depending on (I) the body portion: upper limb, spinal, and lower limb, or (ii) the joint involved: wrist–hand, lumbar, and ankle–foot [3].

Customized orthoses present good outcomes in patients, such as comfort and pain reduction [4], but as they are handmade, their quality highly depends on the competence and expertise of the specialist [5]. Additive manufacturing (AM) presents several advantages to the time-consuming and laborious conventional fabrication of custom orthoses, such as plaster casting [3,6]. The production of the orthoses is faster; the patient’s experience is more comfortable since scanners can be used to aid in obtaining the desired geometry; the number of technicians and the manual work is reduced; the model of the orthoses can be archived and reproduced when necessary; and there is less need for production equipment, therefore less storage space [7].

Fused deposition modelling (FDM) is an AM technology that allows the production of three-dimensional objects through the extrusion of a material layer by layer. This technique allows for high precision in creating complex geometries that are challenging to achieve with traditional methods. Consequently, it can enhance orthoses’ performance, durability, and modern aesthetics [8,9,10]. Furthermore, FDM offers greater accuracy, ease of use, and cost-effectiveness compared to other AM strategies, such as selective laser sintering [11]. Despite these advantages, the benefits of FDM technology are still underexplored [3]. One major challenge is selecting the right material for orthoses, which must meet various mechanical and physical properties [5,12,13]. No single material can meet all adequate criteria, but the final product should be lightweight, user-friendly, cost-effective, durable, body-compatible, and suitable for its intended use (e.g., rehabilitation or support). Using a hard material or an improper design can result in an uncomfortable or biomechanically incorrect orthosis [1,5,13,14,15].

The primary objective of this study is to evaluate nine polymeric materials printed in two printing directions (horizontal and vertical relative to the base plate) by analysing their compressive, flexural, and tensile properties. The second objective is the development of a three-dimensional finite element model of a real ankle–foot orthosis for simulating its deformation, stress, and strain fields under static loading conditions considering daily usage.

## 2. Materials and Methods

### 2.1. Materials Production

**Figure 1:**
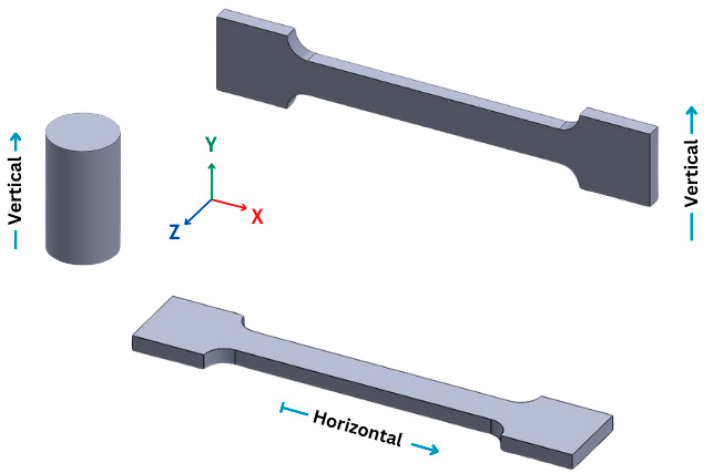
Horizontal (H) and vertical (V) printing directions of the tested specimens.

The study evaluated nine polymeric materials: acrylonitrile butadiene styrene (ABS), Nylon 12, polycarbonate (PC), polycarbonate/acrylonitrile butadiene styrene (PC-ABS), polyethylene terephthalate glycol (PETG), polylactic acid (PLA), thermoplastic polyurethane (TPU), and high-performance polyetherimide (PEI) thermoplastics ULTEM™ 1010, and ULTEM™ 9085. Materials were provided by Stratasys and the specimens were manufactured using a 3D printer by FDM (Stratasys F170 printer, Stratasys, Eden Prairie, MN, USA) with an infill density of 100%, infill angle of 45°, and a slice height of 254 µm. Each material was printed in two directions: horizontal (H) and vertical (V) relative to the base plate, as shown in Figure 1.

### 2.2. Tensile Testing

Tensile tests were conducted following ASTM D638-14 standards [16]. The tested specimens were printed in both H and V orientations. The tests were performed using a universal testing machine (Instron Model 5544, Norwood, MA, USA) equipped with a 100 kN load cell. The test speed was set to 5 mm/min.

### 2.3. Flexural Testing

Flexural tests were conducted using the same universal testing machine, but with a speed test of 2 mm/min according to ISO 178 standard [17]. The specimens used for this test were the same as those used in the tensile test since their specifications correspond to those used for this standard.

### 2.4. Compression Testing

Compression tests were performed according to ASTM D695-23 standards [18]. The cylindrical specimens tested were printed in the V direction. Testing was conducted using the same universal testing machine, with a speed test of 1 mm/min.

### 2.5. Data Analysis of the Mechanical Assays

Tests were conducted at room temperature and for each test type and material, five specimens were tested to ensure statistical reliability. The tensile/flexural/compressive strength, Young’s modulus, and strain at break were recorded for each specimen; the results were averaged, and the standard deviations were calculated. The influence of printing direction on flexural and tensile properties was evaluated on GraphPad Prism 9 software with multiple unpaired *t*-tests. All tests were calculated with a confidence interval of 95%, where statistically significant differences are represented by * *p* < 0.05, ** *p* < 0.01 and *** *p* < 0.001. Correlations for the mechanical assays were also calculated with a Pearson correlation test on GraphPad using the same software and confidence interval above-mentioned.

### 2.6. Static Structural Test

**Figure 2:**
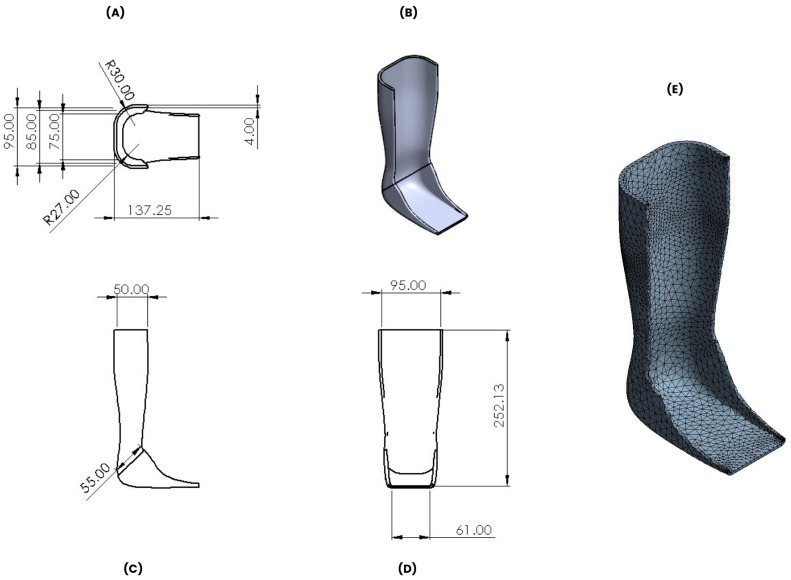
Ankle–foot orthosis design model: Top view (**A**), 3D projection view (**B**), front view (**C**), right view (**D**), and mesh model (**E**).

The static structural analysis of the ankle–foot orthosis was performed for PC, PLA, and ULTEM™ 1010 due to their minimal or low differences in printing direction mechanical results. In this analysis, a real ankle–foot orthosis was simulated. The three-dimensional model was created using SolidWorks 2023, a software from Dassault Systèmes Corporation (Waltham, MA, USA). The model was imported as a Parasolid file (.x_t) into Ansys Workbench 19.2 software (Canonsburg, PA, USA), which provides a common platform integrating various Ansys applications for multi-physics simulations and design optimization. The finite element mesh contained 23,440 nodes and 11,758 elements, the element size was set at 5 mm, and the mesh type was tetrahedral. The physical model and the corresponding assembled meshed can be seen in Figure 2, where different perspective views of the ankle–foot orthosis are shown. The ankle–foot orthosis was designed with an increase in the length of the lever arm and the calf surface area to assure comfort and efficiency [19].

**Figure 3:**
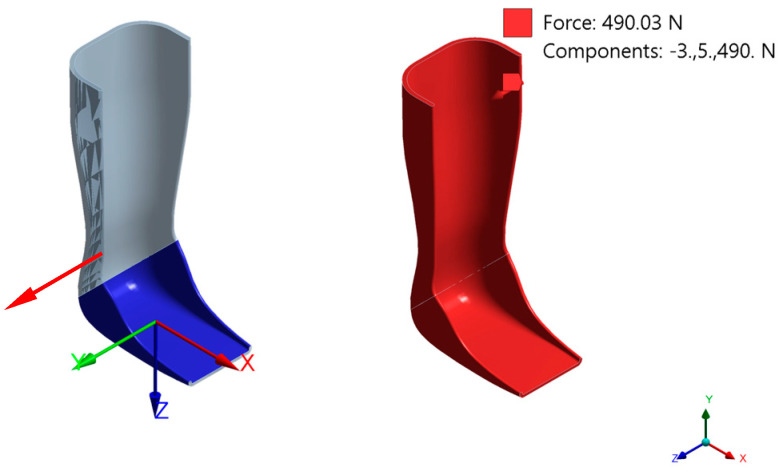
Fixed joint ground to part in blue and applied force in red.

The simulation of the real-life effects can be seen in Figure 3: the area where the foot will be placed was assigned a ground-to-part relation with a fixed joint (in blue); to simulate the contact and force that the body may apply on the ankle–foot orthosis in real life when subjected to static conditions, a force of 490.03 N was used with force vector components (−3, 5, 490) N in X, Y, and Z directions. The force was applied to the entire model as shown in Figure 3. The applied force is according to the investigations of Marques et al. [20] and Ali et al. [21] describing the full contact moment in the gait cycle when the sole fully touches the ground.

The finite element model was assumed to be linear-elastic, homogeneous, and isotropic. The information about the isotropic elasticity, yield, and ultimate strength of the tested materials according to the material’s supplier (Stratasys, Eden Prairie, MN, USA) is shown in Table 1.

A static structural analysis was performed to obtain results relative to the total deformation, equivalent elastic strain, equivalent von Mises stress, and factor of safety defined based on maximum equivalent stress theory and tensile yield. The structural analyses were carried out for the three materials used in the numerical simulations.

## 3. Results

### 3.1. Tensile Tests

**Figure 4:**
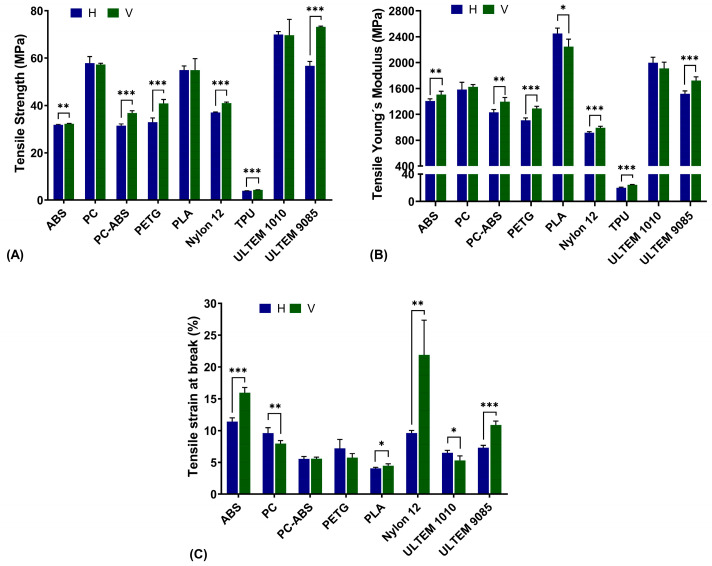
Tensile strength (**A**), Young’s modulus (**B**), and strain at break (**C**) for all materials in both printing directions, horizontal (H) and vertical (V). Statistical analysis was conducted with multiple unpaired *t*-tests, and differences are represented by * *p* < 0.05, ** *p* < 0.01, and *** *p* < 0.001.

The tensile properties of the nine polymeric materials, printed in both H and V directions were evaluated. The three parameters analysed were tensile strength (Figure 4A), tensile Young’s modulus (Figure 4B), and strain at break (Figure 4C). For tensile strength in the H direction, ULTEM^TM^ 1010 gave the highest value of 69.99 ± 1.23 MPa, and TPU was the lowest with a value of 3.97 ± 0.03 MPa. For the V direction, ULTEM™ 8095 gave the highest value with 73.17 ± 0.33 MPa and TPU led to the lowest with a value of 4.36 ± 0.03 MPa. Statistically significant differences were found for most of the materials when comparing the printing directions, such as ABS, PC-ABS, PETG, Nylon12, TPU, and ULTEM™ 9085, where the V direction was the one with the highest values.

For tensile Young’s modulus, TPU was the material with the lowest value for both directions, with a modulus of 20.04 ± 0.93 MPa and 24.18 ± 0.54 MPa for the H and V directions, respectively. The highest values were found in PLA, with a tensile Young’s modulus of 2451.36 ± 81.12 MPa and 2245.74 ± 114.80 MPa, for the H and V direction, respectively. Statistically significant differences were found between directions for the same materials as for tensile strength, where the V direction was the one with the highest values, except for PLA where the H direction gave rise to a higher modulus.

Finally, for strain at break, TPU was the only material that did not lead to a break fracture. PLA was the material with the lower extension with a value of 4.07 ± 0.17% and 4.48 ± 0.31% for the H and V direction, respectively. For the higher values, in the H direction, ABS presented an extension of 11.41 ± 0.60%, and in the V direction, Nylon presented an extension of 21.89 ± 5.46%. Comparing printing directions, all materials led to statistically significant differences, except PC-ABS and PETG. For ABS, PLA, Nylon, and ULTEM™ 9085, the V direction led to higher values, whereas for PC and ULTEM™ 1010, the maximum values were found in the H direction.

### 3.2. Flexural Tests

**Figure 5:**
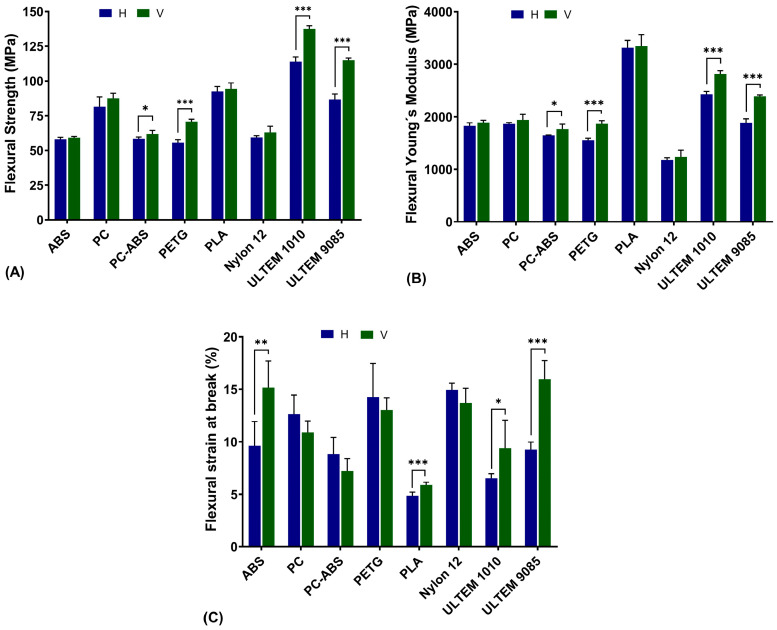
Flexural strength (**A**), Young´s modulus (**B**), and strain at break (**C**) for all materials in both printing directions, horizontal (H) and vertical (V). Statistical analysis was conducted with multiple unpaired *t*-tests, and differences are represented by * *p* < 0.05, ** *p* < 0.01, and *** *p* < 0.001.

Similar to tensile testing, the flexural strength (Figure 5A), flexural Young’s modulus (Figure 5B), and flexural strain at break (Figure 5C) were evaluated for the nine tested materials in both H and V printing directions. It should be noted that in this research it was not possible to evaluate the TPU in our equipment due to its high flexibility, which led to some instability issues resulting in very unreliable graphs.

For the flexural strength in the H direction, the highest value was attributed to ULTEM™ 1010 with a strength of 114 ± 3.27 MPa, and the lowest value to PETG with a value of 55.56 ± 2.26 MPa. In the V direction, ULTEM™ 9085 led to the highest value of 115 ± 1.44 MPa, and ABS exhibited the lowest value with 59.18 ± 0.92 MPa. Focusing on printing direction, statistically significant differences were found for the PC-ABS, PETG, ULTEM™ 1010, and ULTEM™ 9085, where the V direction gave origin to higher values.

For flexural Young’s modulus, in both printing directions, PLA gave the highest values, whereas Nylon gave the lowest values: PLA-H was 1181.00 ± 39.36 MPa, PLA-V was 1236.32 ± 127.28 MPa, Nylon-H was 3313.68 ± 142.03 MPa, and Nylon-V was 3343.27 ± 219.58 MPa. Between directions, the statistically significant differences were similar to flexural strength, where PC-ABS, PETG, ULTEM™ 1010, and ULTEM™ 9085 gave rise to higher values in the V direction.

Strain at break was lower for PLA, in both printing directions, with values of 4.86 ± 0.33% for the H direction, and 5.89 ± 0.26% for the V direction. Nylon led to an extension of 14.93 ± 0.36%, being the material with the higher value in the H direction, whereas the in the V direction was ULTEM™ 9085 with a value of 15.98 ± 1.77%. Once again, TPU also did not lead to a fracture. Between printing directions, statistically significant differences were found for ABS, PLA, ULTEM™ 1010, and ULTEM™ 9085, where the V direction was the one with the higher values.

### 3.3. Compressive Tests

**Figure 6:**
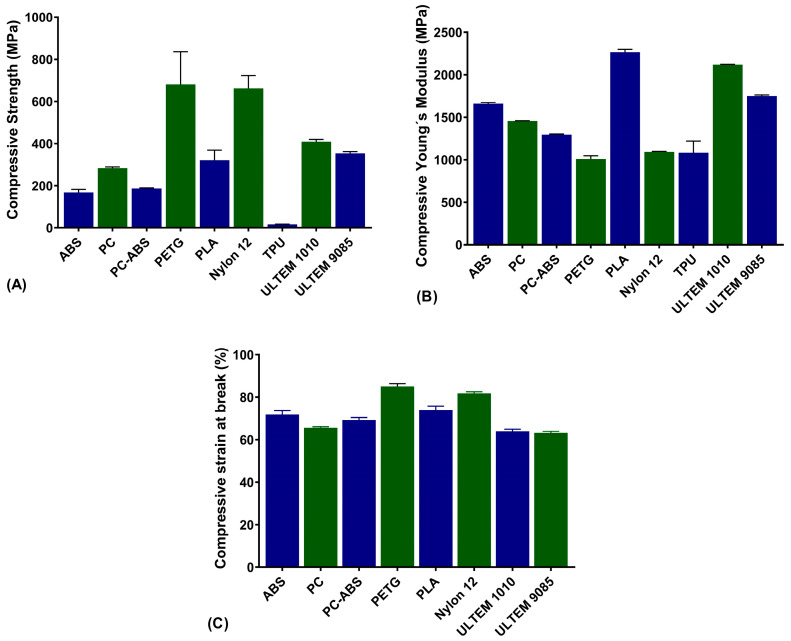
Compression strength (**A**), Young´s modulus (**B**), and strain at break (**C**) for all materials.

Compression properties were only evaluated in the V direction. The values of compressive strength, compressive Young’s modulus, and compressive strain at break obtained in the tests are displayed in Figure 6A, Figure 6B and Figure 6C, respectively.

As far as the compression strength is concerned, the material that led to higher values was PETG with 680.7 ± 155.1 MPa and the lower was TPU with 16.9 ± 1.1 MPa. For compression Young’s modulus, PLA had the higher values with 2264.0 ± 34.0 MPa, and PETG exhibited the lower value with 1008.8 ± 38.3 MPa. For compressive strain at break, the material that had a higher strain value was PETG with 85.1 ± 1.3%, while ULTEM^TM^ led to the lower compressive strain at break with 63.2 ± 0.7 MPa.

### 3.4. Correlation Assays

**Figure 7:**
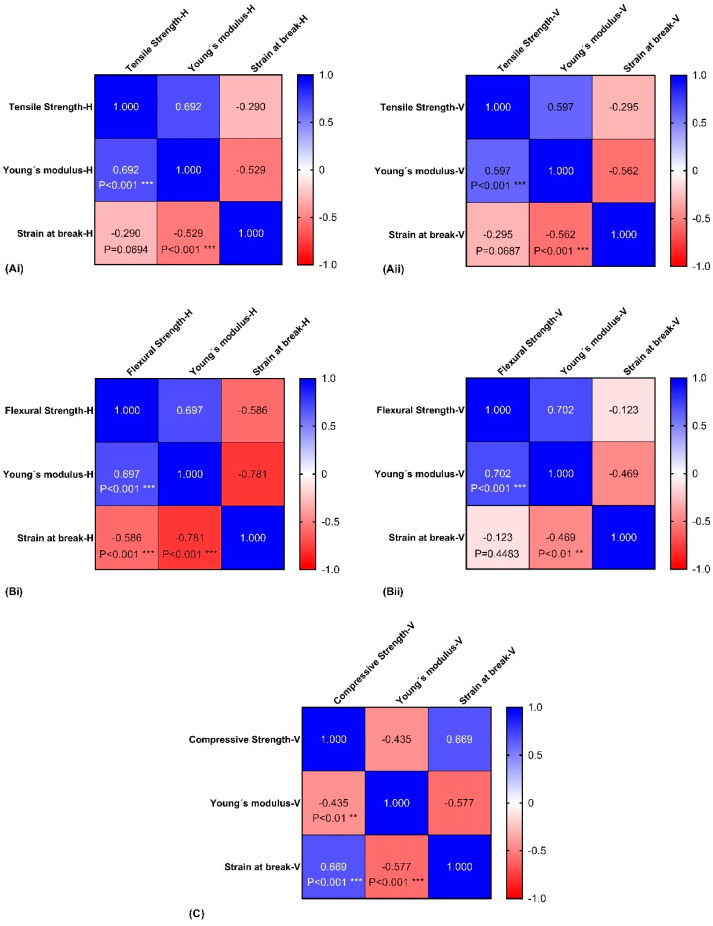
Correlation matrix for each mechanical assay: tensile test in the vertical (**Ai**) and horizontal direction (**Aii**), flexural test in the vertical (**Bi**) and horizontal direction (**Bii**), and compression test (**C**). The correlation coefficient is presented, as well as statistical differences by ** *p* < 0.01 and *** *p* < 0.001.

Analysing the correlation studies for the tensile tests (see Figure 7(Ai,Aii)), it is possible to conclude that the strain at break is not associated with the tensile strength for both printing directions as the correlation coefficients are close to 0 (r = −0.2900 and r = −0.2946, for H and V direction, respectively). On the contrary, the tensile Young´s modulus, showed a negative correlation with the tensile strain at break for both printing directions (r = −0.529 and r = −0.562 for H and V direction, respectively), meaning that when the tensile Young´s modulus increases, the strain at break decreases. This was also confirmed by the *p* < 0.001 which confirmed that this negative correlation is not due to random sampling. As for tensile strength versus tensile Young´s modulus, a positive correlation was found (r = 0.602, and r = 0.597 for H and V direction, respectively, and *p* < 0.001), meaning that when one parameter increases the other also increases.

Regarding the flexural tests (see Figure 7(Bi,Bii)), similar results with the tensile assays were found for the V direction. Flexural strength versus flexural strain at break presented no relationship (r = −0.123, NS), flexural Young´s modulus versus flexural strain at break presented a negative correlation (r = −0.469, *p* < 0.01), and flexural strength versus flexural Young´s modulus presented a positive correlation (r = 0.702, *p* < 0.001). As for the H direction, interestingly, flexural strength versus flexural strain at break presented a negative correlation (r = −0.586, *p* < 0.001). The remaining analyses were similar to the V direction, as flexural Young´s modulus versus flexural strain at break presented a negative correlation (r = −0.781, *p* < 0.001), despite being a much stronger correlation with value close to −1. Finally, flexural strength versus flexural Young´s modulus also presented a positive correlation (r = 0.697, *p* < 0.001).

For compression assays (see Figure 7C), similar to the other mechanical tests, compressive Young’s modulus presented a negative association with compressive strain at break (r = −0.577, *p* < 0.001). The differences were found for the remaining correlations. Compressive strength versus compressive strain at break presented a positive correlation (r = 0.669, *p* < 0.001) and compressive strength versus compressive Young´s modulus presented a negative correlation (r = −0.435, *p* < 0.01).

### 3.5. Static Structural Analysis

**Figure 8:**
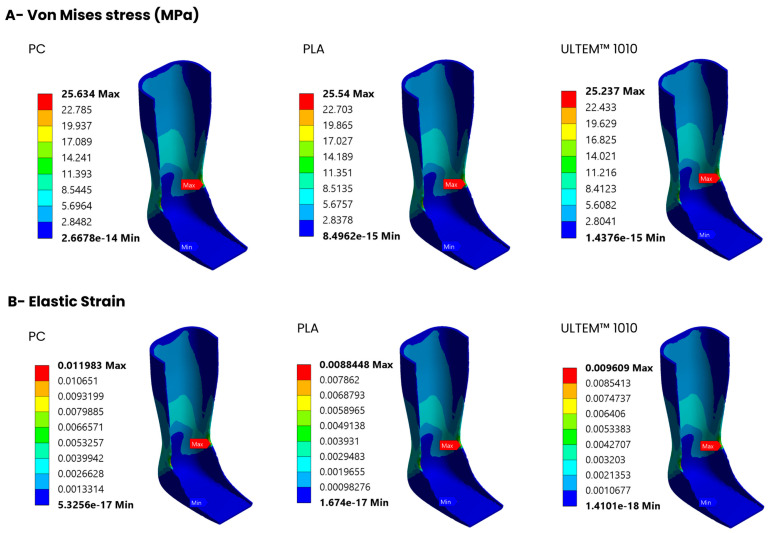

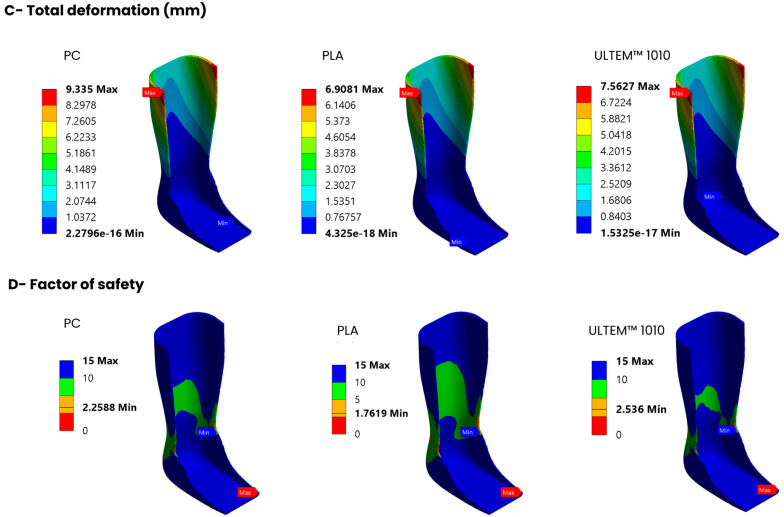
Static structural analysis of the ankle–foot orthosis: (**A**) Equivalent von Mises stress; (**B**) equivalent strain; (**C**) total deformation, and (**D**) safety factor for the three tested materials (PC, PLA, and ULTEM™ 1010).

Static structural analysis was carried out for PC, PLA, and ULTEM™ 1010 due to their minimal or low differences in mechanical results between printing directions, and also because they are the materials with the highest mechanical properties of all the materials, making them suitable for the production of orthotics. The results are represented in colour varying from blue to red, which correspond from the lower to the higher values of the plotted variable. For each material, equivalent stresses (Figure 8A), equivalent strains (Figure 8B), total deformation (Figure 8C), and safety factors (Figure 8D) are presented. Regarding stresses, for the three materials (PC, PLA, and ULTEM™ 1010), the maximum von Mises stresses were around 25 MPa and stress concentrations were more located in the area covering the ankle. The elastic strains were also more visible in that area, where PC showed the highest maximum elastic strain, followed by ULTEM™ 1010 and PLA. The upper area of the ankle–foot orthosis showed a significant deformation for the three materials, with red indicating maximum total deformation. PLA showed the lowest deformation compared to ULTEM™ 1010 and PC, while PC showed the highest deformation. All three materials demonstrated a minimum safety factor greater than 1. Among them, ULTEM™ 1010 had the highest safety factor, followed by PC. PLA showed the lowest safety factor. Table 2 summarises the main results obtained in the numerical simulations for the three materials (maximum von Mises stress, maximum elastic strain, maximum total deformation, and minimum safety factor).

## 4. Discussion

For orthotic AM production, it is essential that the chosen materials can withstand distinct mechanical stresses, including those resulting from flexural, compression, and tensile forces. These properties ensure that the orthosis will be durable and reliable for the patient while maintaining its structural integrity and functionality over time. Different authors have investigated different materials for orthotic production such as PC, PC-ABS, ULTEM, PLA, ABS, and PETG [22,23,24,25,26,27,28,29]. However, a consensus on the most suitable material is still debatable.

FDM-manufactured parts are known to be anisotropic due to the specificities inherent to this AM process, including the printing orientation [30,31]. This is why the mechanical properties of printed materials must be addressed in different orientations to achieve the desired results. Camargo et al. showed that the tensile and flexural strength of PLA-graphene material increases with the increase of the infill, while impact energy decreases. An increase in layer thickness also led to higher values in the referred mechanical properties [32]. Moreover, PLA also exhibited varying flexural strengths depending on the type of filling, such as rectangular, triangular, and honeycomb [33].

In the V direction, the layers of the printed materials are aligned parallel to the loads, while in the H direction, they are aligned perpendicularly. This characteristic resulted in better mechanical performance in the flexural tests, for all the analysed materials. For the tensile tests, the best performance was associated with the V direction, except for the PLA’s Young´s modulus, and for PC and ULTEM™ 1010 strain at break, where the H direction showed higher values. Various studies have reported that printing directions affect the flexural properties of resins [34,35]. Similar findings have been reported for thermoplastics, aligning with the results found in the present study. The specimens printed parallel to the loads, presented higher flexural strength in ULTEM™ 9085 and ABS [36], and higher tensile strength in ABS [37]. The same was also observed for Nylon and ULTEM™ 9085 tensile strength, tensile Young´s modulus, and tensile strain at break [38,39]. Curiously, in the present study ULTEM™ 1010´s tensile strength and tensile Young´s modulus presented similar results between printing directions, but tensile strain at break was also higher for the H direction [39]. Although PLA [40] and PC [41] presented higher tensile strength values for specimens printed parallel to the loads in other studies, this was not observed in the present study. This discrepancy may occur likely due to variations in printing speed and temperature, which can affect the adhesion between layers and the consistency of the filament diameter and its quality, leading to differences in mechanical performance [6,42]. The rapid cooling from the FDM process can leave behind empty spaces due to a very rapid shrinkage of the material which leads to a deficiency in the adhesion between material layers, leading to residual stresses in the material [30].

The strength–ductility of the materials produced can be more effectively analysed through correlation studies of mechanical properties [43]. It is known that Young´s modulus is defined as the ability of a material to resist deformation [44]. The ultimate strength, used in this study as tensile/flexural/compression strength, is the maximum value that an object can resist without breaking [45], and strain at break is the point the material fractures [46]. Results of correlation demonstrated that there is no association between tensile strain at break and tensile strength. This means that the material´s ability to withstand stress in both printing directions does not predict its elongation. As for tensile Young´s modulus and tensile strain at break, there is a negative correlation. This means that materials with a higher Young´s modulus (stiffer materials) are often more brittle. Tensile strength versus tensile Young´s modulus presented a positive correlation, as both are related to the material´s ability to bear loads. For flexural tests, the same conclusions can be drawn, as similar results were obtained. The only difference was found in the H direction, where a negative correlation was found between flexural strength and flexural strain at break. In this direction, not only does a stiffer disc lead to a brittle material, but so does its load-bearing capacity. Lastly, the failure mechanisms of compressive loads led to differences when compared to the tensile and flexural tests: a positive correlation was found between compressive strength and compressive strain at break, meaning that the materials can withstand higher loads and also elongate more; and negative correlation between compressive strength and compressive Young´s modulus, where a material that can withstand more loads does not necessarily exhibit greater stiffness.

Regarding ankle–foot orthosis manufacturing, Raj et al. highlighted the advantages of using AM to produce ankle–foot orthosis compared to conventional manufacturing [47]. Overall PC, PLA, and ULTEM^TM^ 1010 demonstrated the most promising outcomes. Not only do they present superior mechanical properties, but their consistent results in the different printing directions also make them particularly advantageous for orthosis fabrication due to a higher printing flexibility. Thus, they were selected for the simulations. The simulation of the ankle–foot orthosis designed in this study gives realistic results relative to its mechanical performance under real-world conditions while allowing for reduction of the amount of prototype iterations for validation. The results of static structural analysis give an insight into the mechanical performance of the ankle–foot orthoses produced using three distinct materials. The stress distribution patterns are comparable to each other allowing us to identify the concentrated high-stress regions where potential failures can occur. The maximum stresses for PC, PLA, and ULTEM™ 1010 are below their tensile yield strengths which ensures that the material behaves predictably, within its safe operating limits. PC showed the highest maximum elastic strain, which means that it is prone to deform more compared to ULTEM™ 1010 and PLA. The total deformation results show that PC is prone to significant deformation while PLA has a lower deformation, whereas ULTEM™ 1010 balances between them. Regarding the safety factor, a value lower than 1 indicates potential failure. In the three cases, the safety factor is higher than 1, which indicates that these materials are in the acceptable range. The safety factors of PC and ULTEM™ 1010 are greater than 2, indicating that the model can handle twice the force applied without failing. Based on the simulation results, it is clear that the current orthoses design will experience high stress levels in specific areas, regardless of the material used.

## 5. Conclusions

Through these tests, we can select materials that will optimize the performance of orthoses, contributing to better patient outcomes and satisfaction. The present study led to a better understanding of nine polymeric materials under various mechanical conditions. The correlation studies emphasized the importance of considering different mechanical properties for evaluating material performance. PC, PLA, and ULTEM^TM^ 1010 presented the most interesting results because there were no differences in values between the print directions, making them more advantageous for orthosis printing. This led to the choice of a virtual ankle–foot orthosis based on these three materials. The FEA of the ankle–foot orthosis gives insight into the mechanical behaviour of an ankle–foot orthosis under static conditions. This result from the static structural analysis can help in optimizing ankle–foot orthoses for better performance under real loading conditions. Based on the numerical simulations, ULTEM^TM^ 1010 exhibited the best performance.

## Figures and Tables

**Table 1:** Material data.

Material	Young’s Modulus (MPa)	Poisson’s Ratio	Tensile Yield Strength (MPa)	Tensile Ultimate Strength (MPa)
PC	2250	0.39	57.9	57.3
PLA	3039	0.39	45.0	48.0
ULTEM™ 1010	2770	0.36	64.0	81.0

**Table 2:** Result summary for the PC, PLA, and ULTEM™ 1010.

Material	Maximum Von Mises Stress (MPa)	Maximum Elastic Strain (mm/mm)	Maximum Total Deformation (mm)	Minimum Safety Factor
PC	25.63	11.98 × 10^−3^	9.34	2.26
PLA	25.54	8.85 × 10^−3^	6.91	1.76
ULTEM™ 1010	25.24	9.61 × 10^−3^	7.56	2.54

## Data Availability

The original contributions presented in the study are included in the article; further inquiries can be directed to the corresponding author/s.
